# Is Early Preventive Caffeine Safe and Effective in Premature Neonates? A Clinical Trial

**DOI:** 10.1155/2022/8701598

**Published:** 2022-06-02

**Authors:** Negar Sajjadian, Peymaneh Alizadeh Taheri, Mahboobeh Jabbari

**Affiliations:** ^1^Tehran University of Medical Sciences, Shariati Hospital, Tehran, Iran; ^2^Tehran University of Medical Sciences, Bahrami Children Hospital, Tehran, Iran

## Abstract

**Background:**

Advantages of caffeine for the treatment of apnea of prematurity (AOP) have prompted clinicians to use it as a preventive drug even before the occurrence of apnea.

**Objective:**

To compare the effect of early preventive caffeine therapy with routine late preventive caffeine on the occurrence rate of apnea of prematurity, bronchopulmonary dysplasia (BPD) and related radiographic changes, necrotizing enterocolitis (NEC), intraventricular hemorrhage (IVH), and patent ductus arteriosus (PDA), the need for mechanical ventilation, the length of mechanical ventilation, and the length of hospitalization.

**Materials and Methods:**

In this open randomized clinical trial study, 90 preterm neonates with the gestational age of 25-35 weeks were divided into 2 groups: group A received caffeine during the first two days of life (early preventive caffeine), while group B received caffeine during the third to the tenth day of life (late preventive caffeine). The occurrence rate of AOP and other outcomes were the primary outcomes. The adverse effects of caffeine in each group were the secondary outcomes.

**Results:**

The total occurrence rate of AOP was significantly higher (32.6%) in the late group versus (6.8%) in the early group (*p* = 0.002). The total occurrence rate of BPD was also significantly higher (37%) in the late group versus (18.2%) in the early group (*p* = 0.047). On the other hand, we found a lower need for mechanical ventilation, shorter length of mechanical ventilation, shorter length of hospitalization, and a lower occurrence rate of PDA, NEC, and IVH in the early group that was not significant. No adverse effect of caffeine was observed in each group.

**Conclusions:**

Early preventive caffeine administration was associated with a significantly lower occurrence rate of AOP, BPD, and BPD radiologic changes. As other outcomes occurred lesser in the early group that were not significant, future studies with more participants are recommended. This study has been registered at the Iranian Registry of Clinical Trials (IRCT20160827029535N8).

## 1. Introduction

Apnea of prematurity is one of the most common diagnoses in the NICU [1]. AOP has been defined as cessation of breathing for ≥20 seconds or a shorter duration accompanied by oxygen desaturation and/or bradycardia or pallor in infants with a GA below 37 weeks [1, 2].

AOP is a developmental delay in preterm infants with lower gestational age (GA). It is partly due to the physiological immaturity of the central nervous system, in particular poor myelination of the immature brainstem that improves spontaneously as GA increases [2, 3].

The occurrence rate of AOP increases with GA and birth weight. AOP occurs in 7% of premature neonates with GA 34–35 weeks to nearly all neonates with GA < 29 weeks or birth weight less <1,000 g [4, 5].

Apnea occurs most often (50%–75%) as a mixed type. Two other types of apneas include a central and an obstructive type, each one with an incidence rate of 10%–25% of cases [1, 6]. In central apnea, airflow ceases in the absence of respiratory effort. In obstructive apnea, there is no airflow, even if the infant attempts to breathe throughout the apnea. The obstruction is due to a combination of passive pharyngeal collapse and laryngeal closure [7]. The mixed apnea starts with a central apneic phase, followed by an upper airway obstruction that increases the desaturation and bradycardia [7, 8]. But in some cases, airway obstruction can occur first [9].

Caffeine and other methylxanthines have been used to treat AOP for the past 40 years [10, 11].

Methylxanthines are the most commonly used medications for the treatment of AOP [12–14]. Theophylline is not routinely used anymore because it has a narrower therapeutic margin to toxicity than caffeine and induces tachycardia and gastrointestinal intolerance. Caffeine citrate is preferred because it has a longer half-life, lack of necessity for plasma level measurement, and more response rate [15] The CAP trial and its subsequent reports of outcomes did not reveal any significant short- or long-term adverse effects of caffeine therapy in the NICU [16, 17].

The effectiveness and safety of caffeine therapy were first studied by Erenberg et al. as well as the international trial on caffeine for apnea (CAP trial) [16–18].

Caffeine is among the most commonly prescribed medications in neonatal intensive care units, and it has now largely replaced other methylxanthines. Caffeine reduces the frequency of apnea, intermittent hypoxemia, facilitates extubating from mechanical ventilation, and reduces the incidence of BPD and PDA in preterm infants. There are controversies regarding the safety and efficacy of high-dose, early vs. late administration, duration of therapy, value in older gestational age infants, and the value of therapeutic drug monitoring [19].

The goal of our study was to compare the effect of early administration of preventive caffeine with late preventive prescription of caffeine on the occurrence rate of AOP, BPD and related radiographic changes, PDA, NEC, and IVH, the requirement for mechanical ventilation, the length of mechanical ventilation, and the length of hospitalization.

## 2. Methods

A total of 100 preterm neonates hospitalized in the NICU of Shariati Hospital, Tehran, Iran, with a gestational age of ≤35 weeks and ≤1500 g were enrolled in the study from 2015 to 2017. The number of participants was determined by a 2-sided alpha 0.05, beta equal to 20%, P1 equal to 0.02, P2 equal to 0.03, level of confidence equal to 95%, a power of 80%, and treatment response rate based on Taha et al.'s study [20].

Newborns' gestational age, gender, birth weight, first-minute Apgar score, fifth-minute Apgar score, mode of delivery, using mechanical ventilation, duration of mechanical ventilation, and length of NICU stay were recorded. The primary outcome of this study was the occurrence rate of AOP, BPD, BPD radiographic changes, NEC, PDA, and IVH, the need for mechanical ventilation, the length of mechanical ventilation, and the length of hospitalization in each group. The adverse effects of caffeine (including tachycardia, dysrhythmia tachycardia, dysrhythmia, feeding intolerance, GER, jitteriness, irritability, and seizures) in each group were the secondary outcome [19].

### 2.1. Subjects

A total of 100 preterm infants with a gestational age of 25-35 weeks (29.01 ± 3.011 weeks) were recruited and randomly allocated to either of the study group using a computer-generated list of random numbers in a 1 : 1 ratio. Fifty (50%) of 100 patients were boys and fifty (50%) were girls. The infants in the early caffeine group received preventive caffeine citrate during the first 48 hours of life. The late caffeine group received preventive caffeine citrate on the third to the tenth day of life. The participants, the caregivers (including the clinicians, the nurses, and mothers) who took care of the neonates, the researcher who collected the data, and the statistician who analyzed them were blinded to the allocation.

The preterm neonates with the gestational age of ≤35 weeks and ≤1500 g were included in the study, but those with any underlying condition (e.g., gastrointestinal anomalies and neurological disorders) and history of receiving any maternal or neonatal relaxant or sedative medications before intervention were excluded from the study because they had risk factors that increased the occurrence rate of AOP in addition to prematurity. If the baby was in the late caffeine arm and developed AOP in the first three days of life, treatment with caffeine was started and the patient was excluded from the study too.

### 2.2. Ethical Considerations

The details of the study protocols were approved by the ethical committee at Tehran University of Medical Sciences (IR.TUMS.MEDICINE.REC.1398.644). This study has been registered at the Iranian Registry of Clinical Trials (IRCT20160827029535N8). Written informed consent was obtained from the parents of patients who participated in this study.

### 2.3. Intervention

Neonates who met the inclusion and exclusion criteria were randomly assigned to an open randomized clinical trial with preventive caffeine that was given during the first two days of life (early caffeine) in group A or during the third to the tenth day of life (late caffeine) in group B as routine or conventional group (control group) due to some NICUs' protocol including our NICU protocol that the preterm neonates routinely receive preventive caffeine at ≥3 days of life.

The caffeine citrate was given with a loading dose of 20 mg per kilogram of bodyweight that was followed by a daily maintenance dose of 5 mg per kilogram in both groups until the infant reached 36 weeks of corrected gestational age. The neonatologist researcher filled the demographic data (Table 1) before intervention, examined the neonates since their birth time, and recorded the data of participants' responses to intervention and the adverse effects of caffeine during intervention. The second researcher gathered the data and filled the SPSS.

The occurrence rate of AOP, BPD, BPD radiographic changes, NEC, PDA, and IVH, the need for mechanical ventilation, the length of mechanical ventilation, and the length of hospitalization were considered as the primary outcomes, and the adverse effects of caffeine were defined as the secondary outcomes. AOP was defined as cessation of breathing for ≥20 seconds or a shorter duration accompanied by oxygen desaturation and/or bradycardia or pallor in infants with a GA below 37 weeks [1, 2]. The occurrence of apnea was recorded in cardiorespiratory monitors plus nursing documentation and was approved clinically at the same time by the fellowship at night time or the neonatologist during the day. BPD was defined as oxygen requirement at 36 weeks postmenstrual age in infants born with birth weight < 1,500 g according to Vermont-Oxford Network [21]; BPD radiographic changes were diagnosed by the neonatologist and the expert radiologist. NEC and IVH of any degree were diagnosed by the neonatologist and the expert radiologist too. The occurrence rate of PDA was evaluated by be echocardiography before intervention, one week after intervention, and at the end of intervention.

### 2.4. Data Analysis

The data were analyzed with SPSS for Windows 21.0 (SPSS Inc., Chicago, IL, USA). Categorical and continuous variables are presented as percentage and mean ± standard deviation (SD), respectively. Comparison of quantitative data between the two groups was performed with an independent sample *t*-test. Chi-square and Fisher's exact test were used for categorical data, and odds ratio and confidence interval were reported. The significance level and power of the study were determined as 5% and 80%, respectively.

## 3. Results

**Figure 1:**
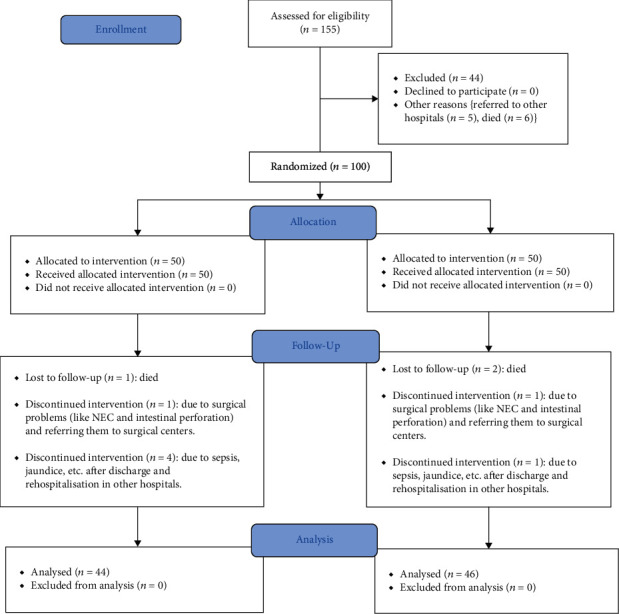
CONSORT flow diagram.

In this open randomized clinical trial, 100 preterm newborns were enrolled. All participants were evaluated by the attending neonatologist. A total of 10 patients including six patients in group A and four patients in group B discontinued intervention due to referring them to surgical centers because of acute surgical problems (e.g., intestinal perforation) or rehospitalization in other hospitals after discharge due to sepsis, jaundice, etc. (Figure 1). A total of 90 preterm newborns with the mean birth weight of 1079.17 ± 232.907 g (690-1500 g) and the mean age of 29.01 ± 3.011 weeks (25-35 weeks) followed the study including forty-four neonates in group A and forty-six neonates in group B. There was no significant difference between sex, birth weight, delivery route, history of chorioamnionitis, and the Apgar score of the first and fifth minute of life between the two groups (Table 1).

The total occurrence rate of AOP following the early preventive caffeine was significantly lower than the late caffeine group (Table 2). The total occurrence rate of BPD and BPD radiographic changes was also significantly lower in the early group (Table 2). The mean length of hospital stays was 18.68 ± 13.006 days in the early group versus 23.27 ± 15.58 days in the late group (*p* = 0.082), and the mean duration of mechanical ventilation was 5.142 ± 3.52 days versus 11.68 ± 6.91 days in the late group (*p* = 0.131). Although the mean length of hospital stays and the mean duration of mechanical ventilation were shorter in the early group, the differences were not significant. On the other hand, we found a lower need for mechanical ventilation and a lower occurrence rate of NEC, PDA, and IVH in the early group that neither difference was significant (Table 2). No adverse effect of caffeine (including tachycardia, dysrhythmia, feeding intolerance, gastroesophageal reflux, jitteriness, irritability, and seizures) was observed in each group.

## 4. Discussion

The present randomized clinical trial was conducted to compare the effectiveness of early preventive caffeine with late preventive caffeine on the occurrence rate of AOP, BPD, BPD radiographic changes PDA, NEC, and IVH, the need for mechanical ventilation, the length of mechanical ventilation, and the length of hospitalization in premature neonates.

According to our recent search (in Medline, PubMed, Ovid, the Cochran Library, Google, and Google Scholar); the previous systematic reviews of Henderson-Smart and De Paoli [22], Park et al. [23], and Kua and Lee [24]; and the recent systematic reviews of Alhersh et al. [25] and Moschino et al. [26], most of the studies about early caffeine administration in preterm infants are retrospective cohort studies like Patel et al. [27], Lodha et al. [15], Taha et al. [20], Feng et al. [28], Bhatt-Mehta et al. [29], Mürner-Lavanchy et al. [30], and Shenk et al. [31], or prospective cohort studies like Borszewska-Kornacka et al. [32] and Du et al. [33].

Almost all previous retrospective or prospective cohort studies have surveyed the effect of early caffeine administration in (1) early clinical outcomes of preterm neonates including BPD, PDA, IVH, NEC, morbidity, and death during neonatal and early infancy and (2) late clinical outcomes including neurodevelopmental disability at 11-18 months and visuomotor, visuoperceptual, and visuospatial abilities at age 11 years.

There are few clinical trials that have compared the preventive effect of caffeine on AOP and neonatal outcomes, including the following:

1. In 2010, Davis et al. studied on 2006 preterm neonates with the birth weight of 500-1250 g randomized in two groups of early or late (<3 days versus ≥3 days) to receive either caffeine citrate (*n* = 1006) or normal saline placebo (*n* = 1000) to treat apnea, prevent apnea, or facilitate extubating. Their study showed that in each subgroup of caffeine used, earlier initiation of caffeine may be associated with a greater reduction in the time of ventilation. Moreover, infants receiving respiratory support derived more neurodevelopmental benefits from caffeine than infants not receiving support [34].

Davis et al. have surveyed the effectiveness of caffeine in comparison with placebo between two times of early days (0–2 days) and late days (3–10 days) similar to our study. Davis et al. have studied on the duration of ventilation and more neurodevelopmental benefits from caffeine in infants receiving respiratory support, while we considered numerous neonatal outcomes including BPD, BPD radiographic changes, PDA, NEC, IVH, the length of mechanical ventilation, the length of hospitalization in premature neonates, and the need for mechanical ventilation in our study.

(2) In 2016, Armanian et al. compared earlier preventive initiation of caffeine (during the first 10 days of life) with a normal saline placebo in premature newborns with the birth weight of ≤1200 g. Twenty infants were enrolled in each group. In the caffeine group, apnea, bradycardia, and cyanosis occurred significantly lesser than in the placebo group. The incidence of IVH, PDA, and NEC was similar in both groups, but the BPD occurred significantly lesser in the caffeine group. No side effect (tachycardia) was reported in the caffeine group [35].

Armanian et al. have surveyed the effectiveness of caffeine in comparison with placebo during the first ten days of life, but our study has compared the effectiveness of preventive caffeine administration between two times of early days (0–2 days) and late days (3–10 days). Armanian et al. have compared the occurrence rate of apnea, bradycardia, cyanosis, IVH, PDA, NEC, and BPD between caffeine and placebo group, while we considered numerous neonatal outcomes including BPD, BPD radiographic changes, PDA, NEC, IVH, the length of mechanical ventilation, the length of hospitalization in premature neonates, and the need for mechanical ventilation in our study.

(3) Schmidt et al. performed some clinical trial studies randomly assigned premature neonates with birth weights of 500 to 1250 g to receive either caffeine or placebo for prevention or treatment of apnea and the facilitation of the removal of an endotracheal tube during the first 10 days of life until therapy for apnea of prematurity in 2006, 2007, 2012, and 2017.
  - In 2006, Schmidt et al. assigned 963 premature infants to their study. At a postmenstrual age of 36 weeks, the placebo group received significantly more supplemental oxygen than the caffeine group. Positive airway pressure was discontinued one week earlier in the caffeine group. The rates of death, ultrasonographic signs of brain injury, and NEC did not differ significantly between the two groups. Caffeine administration reduced the rate of PBD in very low birth weight infants [16]
  - In 2007, Schmidt et al. enrolled 937 premature infants in their study. The rate of death or survival with a neurodevelopmental disability was significant. Treatment with caffeine as compared with placebo declined significantly the rate of cerebral palsy and of cognitive delay. The rates of death, deafness, and blindness and the growth of weight, height, and head circumference at follow-up did not differ significantly between the two groups. Caffeine could improve the rate of survival without neurodevelopmental disability at 18 to 21 months in these infants [17]
  - In 2012, Schmidt et al. performed a five-year follow-up in 31 academic hospitals on preterm infants who had been enrolled in the randomized, placebo-controlled Caffeine for Apnea of Prematurity. The data of 1640 premature neonates were available for evaluation of the main outcome at 18 months of age and at 5 years. The different rates of death, motor impairment, behavior problems, poor general health, deafness, and blindness were not significant between the two groups. The incidence of cognitive impairment was lower at 5years than at 18 months, but it was not significant too. The rate of death or disability did not differ significantly between the caffeine group and placebo group [36]
  - In 2017, Schmidt et al. conducted a follow-up study at 14 academic hospitals on preterm infants who had been enrolled in the randomized, placebo-controlled Caffeine for Apnea of Prematurity. A total of 1202 preterm infants were eligible for this study of whom 920 (76.5%) with median age of 11.4 years had adequate data for follow-up. Caffeine therapy did not significantly decline the combined rate of academic, functional, and behavioral impairments but reduced the risk of motor impairment in 11-year-old children with very low birth weight. Neonatal caffeine therapy was effective and safe into middle school age in this trial [37].

There are three main differences between our study and the mentioned clinical trials including the following:

- First, the mentioned studies of Schmidt et al. in 2006, 2007, 20012, and 2017 have not identified the number of patients in each group of patients receiving caffeine for prevention or treatment of apnea or the facilitation of the removal of an endotracheal tube, so the results belong to all three groups and the rate of responsiveness in preventive groups is not identified
- Second, the mentioned studies have surveyed the effectiveness of caffeine in comparison with placebo during the first ten days of life, but our study has compared the effectiveness of preventive caffeine administration between two times of early days (0–2 days) and late days (3–10 days)
- Third, in opposite to the mentioned clinical trial studies, our study has compared the occurrence rate of AOP and numerous neonatal outcomes including BPD, BPD radiographic changes, PDA, NEC, IVH, the length of mechanical ventilation, the length of hospitalization in premature neonates, and the need for mechanical ventilation

To the best of our knowledge, our study is the only clinical trial study that has compared the effectiveness of early preventive (0–2 days) with late preventive (3–10 days) caffeine administration in the occurrence rate of AOP in premature neonates with gestational age ≤ 35 weeks and numerous neonatal outcomes including BPD, BPD radiographic changes, PDA, NEC, IVH, the length of mechanical ventilation, the length of hospitalization in premature neonates, and the need for mechanical ventilation

(4) Katheria et al. compared the effects of early and late (routine) initiation of caffeine in nonintubated preterm neonates. A total of 21 neonates < 29 weeks of gestational age were randomized to receive intravenous caffeine citrate or placebo either before 2 hours of age (early) or at 12 hours of age (routine). There was no difference in the need for intubation or vasopressors by 12 hours of age. Early caffeine was associated with improved blood pressure and systemic blood flow. Heart rate, left ventricular output, and stroke volume were not significantly affected. Cerebral oxygenation transiently decreased 1 hour after caffeine administration. There were no differences in other outcomes. In this pilot study, they found that early caffeine administration was associated with improved hemodynamics and suggested larger studies to determine whether early caffeine reduces intubation and related long-term outcomes [38].

Katheria et al. compared only the effects of early (before 2 hours of age) and late (at 12 hours of age = routine) initiation of caffeine in nonintubated preterm neonates on hemodynamic improvement and no other early or late outcomes.

(5) Skouroliakou et al. compared standard doses of theophylline and caffeine for apnea of prematurity randomly received either theophylline or caffeine for treatment or prevention of apnea in seventy neonates less than 33 weeks of gestation. Thirty-seven neonates received theophylline (T) and thirty-three neonates received caffeine (C) for treatment (8 T/10 C) or prevention of apnea (29 T/23 C). Treatment with either methylxanthine significantly decreased apnea events while only C prophylaxis appeared to control apnea in infants at risk. There was a significant decrease in apnea frequency only in the infants receiving caffeine. The benefit of C over T for premature infants with <33 weeks gestation was found only during the first week of therapy with no more benefit after the first week of therapy.

Skouroliakou et al. just studied on the effectiveness of caffeine for treatment or prevention of apnea and no other outcomes [39].

In our study, the total occurrence rate of AOP, BPD, and BPD radiographic changes was significantly lower in the early group. Other neonatal outcomes including PDA, NEC, and IVH occurred lesser, and the need for mechanical ventilation, the length of mechanical ventilation, and the length of hospitalization were shorter in early caffeine administration group, but the differences were not significant. So, future studies with more participants are suggested.

Generally, no obvious adverse effects have been reported after caffeine administration in previous studies unless following accidental overdose or using high-dose caffeine therapy [40, 41].

In our study, there was no adverse effect of caffeine during the intervention in both groups.

## 5. Conclusions

Early preventive caffeine administration was associated with a significant lower occurrence rate of AOP, BPD, and BPD radiologic changes. As other outcomes occurred lesser in early group that were not significant, future studies with more participants are recommended.

## Figures and Tables

**Table 1:** Baseline characteristics of early and late caffeine groups.

Baseline characteristics	Early caffeine group (*n* = 44)	Late caffeine group (*n* = 46)	P-value
Sex, *n* (%) Boys	19 (43.2%)	26 (56.5%)	0.206
Gestational age, weeks, mean ± SD	29 ± 3.073	29.02 ± 2.985	0.968
Birth weight, grams, mean ± SD	1048.18 ± 245.23	1108.8 ± 219.049	0.165
Apgar at 1 min, IQR	6 (1)	6 (1)	0.92
Apgar at 5 min, IQR	8 (1)	8 (1)	0.92
Mode of delivery C/S	37 (84.1%)	31 (67.4%)	0.065
Antenatal chorioamnionitis	2(4.5%)	5 (10.8%)	0.263
Antenatal steroids use	14 (46.6%)	22 (47.8%)	0.121
Surfactant use	32 (72.7%)	33 (71.7%)	0.92

SD: standard deviation; IQR: interquartile range (*Q*_3_ − *Q*_1_); C/S: cesarean section.

**Table 2:** Comparison of study outcomes between early and late caffeine groups.

Study outcomes	Early group (*N* = 44)	Late group (*N* = 46)	Odds ratio (95% CI)	*p* value
Apnea of prematurity, *n* (%)	3 (6.8%)	15 (32.6%)	0.151 (0.04-0.569)	0.002
Bronchopulmonary dysplasia, *n* (%)	8 (18.2%)	17 (37%)	0.379 (0.143-0.964)	0.047
Bronchopulmonary dysplasia radiographic changes, *n* (%)	3 (6.8%)	10 (21.7%)	0.301 (0.76-1.196)	0.044
Mechanical ventilation, *n* (%)	23 (52.2%)	31 (67.4%)	0.53 (0.226-1.245)	0.143
Necrotizing enterocolitis, *n* (%)	8 (18.2%)	14 (30.4%)	0.508 (0.189-1.368)	0.176
Patent ductus arteriosus, *n* (%)	16 (36.3%)	18 (39.1%)	0.812 (0.347-1.899)	0.631
Intraventricular hemorrhage, *n* (%)	8 (18.2%)	9 (19.5%)	0.914 (0.317-2.629)	0.867

CI: confidence interval.

## Data Availability

The original contributions presented in the study are included in the article. Further inquiries can be followed by sending their request via the corresponding author's email.

## Additional Points

*Limitations*. Future studies with more participants in this age group are suggested to compare the preventive effect of caffeine in two different times in the first week of life to emphasize the results of this study. There was no control group in our study, so future studies with control group are recommended too. *Innovations and Breakthroughs*. First is comparing the preventive effect of caffeine in two different times in the first week of life. Second is surveying numerous neonatal outcomes in one study.
